# *NUP214* fusion genes in acute leukemias: genetic characterization of rare cases

**DOI:** 10.3389/fonc.2024.1371980

**Published:** 2024-03-20

**Authors:** Marta Brunetti, Kristin Andersen, Signe Spetalen, Andrea Lenartova, Liv Toril Nygård Osnes, Helen Vålerhaugen, Sverre Heim, Francesca Micci

**Affiliations:** ^1^ Section for Cancer Cytogenetics, Institute for Cancer Genetics and Informatics, The Norwegian Radium Hospital, Oslo University Hospital, Oslo, Norway; ^2^ Department of Pathology, The Norwegian Radium Hospital, Oslo University Hospital, Oslo, Norway; ^3^ Institute of Clinical Medicine, Faculty of Medicine, University of Oslo, Oslo, Norway; ^4^ Department of Haematology, Oslo University Hospital, Oslo, Norway; ^5^ Department of Immunology, Oslo University Hospital, Oslo, Norway

**Keywords:** acute leukemias, rare leukemias, cytogenetics, 9q34-NUP214, fluorescence *in situ* hybridization, array comparative genomic hybridization, fusion gene

## Abstract

**Introduction:**

Alterations of the *NUP214* gene (9q34) are recurrent in acute leukemias. Rearrangements of chromosomal band 9q34 targeting this locus can be karyotypically distinct, for example t(6;9)(p22;q34)/*DEK::NUP214*, or cryptic, in which case no visible change of 9q34 is seen by chromosome banding.

**Methods:**

We examined 9 cases of acute leukemia with *NUP214* rearrangement by array Comparative Genomic Hybridization (aCGH), reverse-transcription polymerase chain reaction (RT-PCR), and cycle sequencing/Sanger sequencing to detect which fusion genes had been generated.

**Results:**

The chimeras *DEK*::*NUP214*, *SET*::*NUP214*, and *NUP214*::*ABL1* were found, only the first of which can be readily detected by karyotyping.

**Discussion:**

The identification of a specific *NUP214* rearrangement is fundamental in the management of these patients, i.e., AMLs with *DEK::NUP214* are classified as an adverse risk group and might be considered for allogenic transplant. Genome- and/or transcriptome-based next generation sequencing (NGS) techniques can be used to screen for these fusions, but we hereby present an alternative, step-wise procedure to detect these rearrangements.

## Introduction

Leukemias are a highly heterogeneous - both phenotypically and pathogenetically - group of aggressive neoplasias that affect the production and maturation of bone marrow cells (1, 2). They are classified into two major groups: myeloid leukemias that involve more or less mature granulocytes, erythrocytes, megakaryocytes, and/or monocytes, and the lymphoid ones that involve lymphocytes and their precursor cells (3). Based on disease development (classically) or the degree of cellular maturation (currently), leukemias are additionally classified as acute or chronic. Acute myeloid leukemia (AML) is the most common leukemia in adults (4). Depending on phenotypic features such as cellular morphology, but increasingly in later years on which acquired genetic change brought about neoplastic transformation, the various leukemias are subdivided further into entities with distinctive prognostic features dependent on the therapeutic regimens that experience has shown to be preferable (1). For these reasons, one currently pays more attention to (patho)genetic features when diagnosing and treating leukemia patients; a precise understanding of what lies behind leukemogenesis in individual patients has become adamant in clinical state-of-the-art medicine. To do this, studies of the leukemic cells with different techniques are necessary.

In this study, we selected for examination 9 acute leukemia cases with rearrangements of chromosomal band 9q34 detected either by G-banding and/or FISH, but excluding cases displaying the translocation t(9;22)(q34;q11) that characterizes Philadelphia (Ph)-positive leukemias. We used array comparative genomic hybridization (aCGH), reverse-transcription polymerase chain reaction (RT-PCR), and cycle sequencing/Sanger sequencing to identify the fusion genes generated by the different genomic rearrangements.

## Materials and methods

### Material

Nine acute leukemias, from 6 males and 3 females aged between 8 and 78 years (**Table 1**), were investigated diagnostically in our laboratory during 2011 and 2023. Bone marrow aspirated and/or blood sampleswere found by karyotype/FISH analysis to carry cells with rearrangement of chromosomal band 9q34 other than the t(9;22) specific for Ph-positive leukemias. The study was approved by the Regional Committee for Medical and Health Research Ethics (REK, project number S-0747a; http://helseforskning.etikkom.no.

**Table 1:** Overview of clinical diagnostic parameters and molecular analysis of the nine acute leukemias.

Case nr	Gender/age	Diagnosis	Blasts (%)	Karyotype	Percentage of cells with split of *NUP214* probe	Fusion Transcripts detected
1	M/78	AML	42%	47,XY,t(6;9)(p22;q34),+15[8]	(97%)	*DEK::NUP214* (exon9::exon18)
2	M/62	AML	55%	46,XY, t(6;9)(p22;q34)[15]	(92%)	*DEK::NUP214* (exon9::exon18)
3	M/34	AML	24%	46,XY,t(6;9)(p22;q34)[12]	(93%)	*DEK::NUP214* (exon9::exon18)
4	F/15	AML	36%	46,XX,t(6;9)(p22;q34)[10]	(90%)	*DEK::NUP214* (exon9::exon18)
5	F/55	AML	27%	46,XX,t(6;9)(p23;q34) [10]	(93%)	*DEK::NUP214* (exon9::exon18)
6	M/23	ALAL-NOS	65%	NA	-	*SET::NUP214* (exon7::exon18)
7	F/41	AUL	60%	82~92,inc[cp10]	(70%)	*SET::NUP214* (exon7::exon18)
8	M/23	near ETP-ALL,	97%	46,XY	(28%)	*NUP214::ABL1* (exon31::exon2) *PCM1::FGFR1* (exon36::exon10)
9	M/8	T-ALL	95%	46,XY,del(11)(q14)[8]	(83%)	*NUP214::ABL1* (exon29::exon2)

NA, not available; -, analysis not done; AML, Acute myeloid leukemia; ALL, Acute lymphoblastic leukemia; ALAL, Acute leukemia of ambiguous lineage, not otherwise specified; AUL, Acute undifferentiated leukemia; near ETP-ALL, near early T-cell precursor ALL; M, Male; F, Female.

### G-banding and fluorescence *in situ* hybridization analyses

Hematopoietic cells were short-term cultured, harvested, stained for G-banding analysis, and analyzed cytogenetically as previously described (5, 6). The karyotype description followed the recommendations of the International System of Cytogenomic Nomenclature (7). In addition, routine leukemia-relevant FISH analyses were performed according to standard protocols. For acute myeloid leukemia (AML), tests were performed using commercial probes (Cytocell, Oxford Gene Technology, Begbroke, Oxfordshire, UK) to detect deletions of 5q, 7q, 20q, and the *TP53* locus (17p13), splitting of the *KMT2A* locus (previously called *MLL*; 11q23), as well as for the possible presence of fusion genes *PML::RARA*, *RUNX1::RUNX1T1*, and *CBFB::MYH11.* For acute lymphoblastic leukemia (ALL), FISH investigations (probes from Cytocell, Oxford Gene Technology) aimed to detect deletion of *CDKN2A* (9p21), fusion genes *ETV6::RUNX1* and *BCR::ABL1*, rearrangement of *ABL1* (9q34), *MYC* (8q24), *KMT2A* (11q23.3), *TCF3* (19p13.3), and *IGH* (14q32.33), as well as the possible presence of additional chromosomes typical of a hyperdiploid karyotype.

Additional FISH investigations to detect rearrangement of the *NUP214* locus (9q34) were performed using the Dual Color Break Apart Probe (Zytovision, ZytoLight, Bremerhaven, Germany) on those cases from which suitable material was available. Chromosome preparations were counterstained with 0.2 μg/ml DAPI and overlaid with a 24×50 mm^2^ coverslip. Fluorescent signals were captured and analyzed using the CytoVision system (Leica Biosystems, Newcastle, UK).

### DNA and RNA extraction and sequencing

Fresh frozen bone marrow or blood was used to extract DNA and RNA. DNA was extracted using the Maxwell 16 extractor (Promega, Madison, Wisconsin, United States) and purified using the Maxwell 16 Cell DNA Purification kit (Promega) according to the manufacturer’s recommendations. RNA was extracted using a miRNeasy kit (Qiagen, Hilden, Germany) or the Monarch Total RNA miniprep kit (New England Biolabs, Ipswich, Massachusetts, USA). The concentrations were measured using QIAxel microfluidic UV/VIS spectrophotometer (Qiagen) and Quantus fluorometer (Promega).

### Array comparative genomic hybridization

Array Comparative Genomic Hybridization (aCGH) using CytoSure Consortium Cancer + SNP arrays (Oxford Gene Technology, Oxford, UK) was performed to screen for genomic imbalances. Data were analyzed using Agilent Feature Extraction Software (Agilent Technologies; version 10.7.3.1) and CytoSure Interpret Software (Oxford Gene Technology; version 4.9.40). Copy number aberrations (CNA) were identified using the Circular Binary Segmentation (CBS) algorithm, adding a custom-made aberration filter defining imbalances as a region with a minimum of five gained/lost probes (8). Imbalances were scored according to (9); however, also those smaller than 5Mb were considered. Annotations were based on the human reference sequence GRCh37/hg19 (10).

### Reverse transcription polymerase chain reaction

One µg of total RNA was reverse-transcribed in a 20 µL reaction volume using iScript Advanced cDNA Synthesis Kit for RT-PCR according to the manufacturer’s instructions (Bio-Rad, Hercules, CA, USA). Primers used for PCR reactions are listed in **Supplementary Table 3**. The PCR amplifications were run on a C-1000 Thermal cycler (Bio-Rad) with an initial denaturation at 94°C for 30 sec followed by 35 cycles at 98°C for 7 sec, 60°C for 30 sec, and a final extension at 72°C for 1 min. Three µL of the PCR product were stained with GelRed (Biotium, Hayward, CA, USA), analyzed by electrophoresis through 1.0% agarose gel, and photographed. The remaining PCR products were purified using the MinElute PCR Purification Kit (Qiagen) and sequenced using an Applied Biosystems SeqStudio Genetic Analyzer system with BigDye™ Terminator v1.1 Cycle Sequencing Kit (ThermoFisher Scientific, Waltham, MA, USA) according to the company’s recommendations. The basic local alignment search tool (BLAST) software (BLAST; https://blast.ncbi.nlm.nih.gov/Blast.cgi; accessed on 15 April 2023) was used for computer analysis of sequence data (11). The BLAT alignment tool and the human genome browser at the University of California, Santa Cruz (UCSC) were also used to map the sequences on the Human GRCh37/hg19 assembly (BLAT; http://genome.ucsc.edu/cgi-bin/hgBlat; accessed on 15 April 2023) (12).

### Quantitative RT-PCR analyses

qRT-PCR was carried out to determine the expression level of T-cell leukemia homeobox 3 (*TLX3*) in T-ALL patients using the CFX96 Touch Real-Time PCR detection system (Bio-Rad Laboratories). Reactions were carried out in triplicate using TaqMan Assays and the TaqMan Universal Master Mix II with UNG (Applied Biosystems, Foster City, CA) according to the manufacturer’s protocol. The thermal cycling parameters consisted of 40 cycles of an initial denaturation step of 95°C for 10 minutes, followed by 60°C for 1 min. The primers used for the qRT-PCR reaction were *TLX3* (Hs00253271_m1; ThermoFisher) and, as an endogenous control, actin beta (*ACTB*; Hs01060665_g1, ThermoFisher). Human universal reference total RNA (Clontech, Mountain View, CA) was used as internal reaction control.

## Results

Four of the nine leukemias (cases 2, 3, 4, and 5) had t(6;9)(p22;q34) as a sole aberration by G-banding. Case 1 had trisomy 15 in addition to the t(6;9). Altogether, five of the leukemias were diagnosed as AML (cases 1-5). One acute undifferentiated leukemia (AUL; case 7) showed a complex, near-tetraploid karyotype. Cases 8 and 9, diagnosed as near ETP-ALL and T-ALL, showed a normal karyotype and the chromosomal deletion del(11)(q14), respectively. The karyotype from the ALAL-NOS patient (case 6) was not available. All karyotype descriptions as well as FISH profiles are listed in **Table 1**; **Supplementary Table 1**. The FISH analyses were those recommended by the WHO (1), national (https://www.helsedirektoratet.no/retningslinjer/maligne-blodsykdommer–handlingsprogram), ALLTogether (ClinicalTrials.gov Identifier: NCT04307576), and NOPHO (2008) protocols (13) as standard for the diagnosis of AML and ALL. Signal patterns detected by FISH did not show any aberrations for any probes except for *ABL1* probe (9q34). These FISH data, together with the *NUP214* signal pattern, are summarized in **Table 1**; **Supplementary Table 1**. Mutation analysis and flow cytometry data are shown in **Supplementary Table 1**.

The imbalances detected by aCGH analyses are presented in **Supplementary Table 2**. Genomic imbalances were detected in cases 1, 5, 6, 7, 8, and 9. A total of 69 imbalances were scored (range 1 to 29), with losses being more frequent than gains.

**Figure 1:**
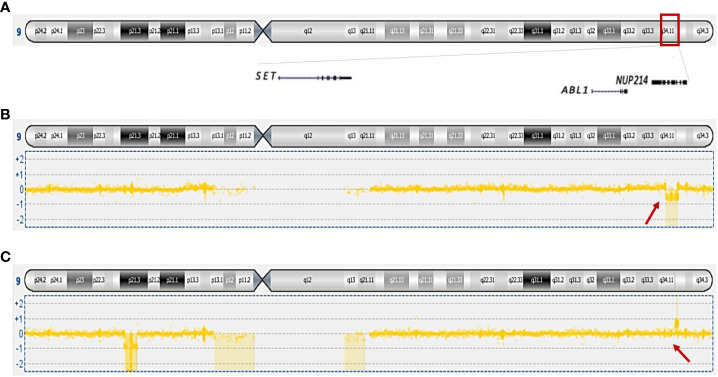
Overview of genes mapping on 9q34 and genomic imbalances scored on this chromosomal band. **(A)** Ideogram of chromosome 9 showing the mapping position of *SET*, *ABL1*, and *NUP214;* **(B)** loss of 33.62 kb genomic material from case 8. **(C)** gain of 97.54 kb genomic material from case 9; in addition, loss of 9p is shown. Arrows are pointing to the imbalances at 9q34.

In cases 2, 3, and 4, no imbalances were seen by aCGH, confirming the balanced rearrangement t(6;9)(p22;q34), seen byG-banding. Cases 1 and 5 showed a similar karyotypic t(6;9); however, these genomes were found to be imbalanced by aCGH (**Supplementary Table 2**). The remaining cases showed imbalances affecting 9q34, the chromosome band where the gene *NUP214* is located demonstrating that cryptic rearrangements undetected by G-banding were present. Of note, cases 6, 7, 8, and 9 showed loss and/or gain of genetic material from 9q33.3-q34.1 less than 5Mb (**Figure 1** **) (** 9 **).** aCGH identified gains on chromosome band 9q34.12-q34.1 (415 Kb) in cases 8 and 9, and losses on 8p22 (415 Kb) and 8p11.23-p11.22 (520 Kb) in case 8. (**Supplementary Table 1**).

**Figure 2:**
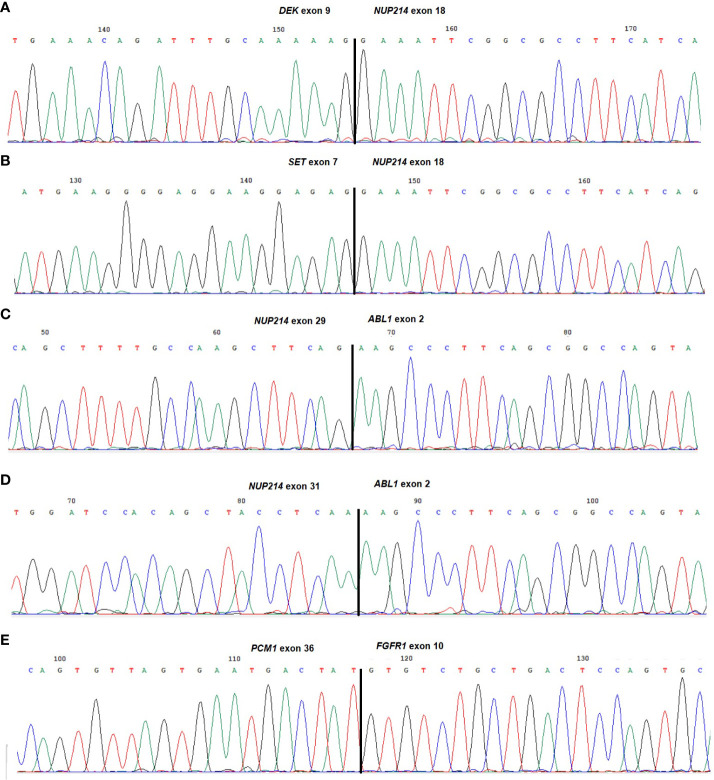
Partial chromatograms of the different chimeric transcripts. **(A)** *DEK*::*NUP214* junction found in cases 1-5; **(B)** *SET*::*NUP214* junction found in cases 6 and 7; **(C)** *NUP214*::*ABL1* junction found in case 8; **(D)** *NUP214*::*ABL1* variant junction found in case 9; **(E)** *PCM1::FGFR1* junction found in case 8. The exons involved in the breakpoints are reported.

A *DEK*::*NUP214* fusion was identified in five leukemias (cases 1-5), whereas *SET*::*NUP214* and *NUP214*::*ABL1* were found in two cases each (cases 6 and 7, and 8 and 9, respectively). Of interest, an additional in-frame fusion was detected between *PCM1* (8p22; exon 36) and *FGFR1* (8p11.23-p11.22; exon 10) in case 8 (**Table 1**; **Figure 2**).

FISH was performed to validate *NUP214*-rearrangements using a break-apart probe (**Table 1**). The expression level of *TLX3* was investigated in the T-ALL cases. The mean quantification cycle (Cq Mean) of *TLX3* was found to be 18.65 and 21.53 for cases 8 and 9, respectively. The Cq Mean for *ACTB* was 18.90 and 22.37 for cases 8 and 9, respectively (both T-ALL); the human reference control gave a Cq Mean of 20.01.

## Discussion

Rearrangements of chromosomal band 9q34 are common and important in the genesis and classification of leukemias (‘https://mitelmandatabase.isb-cgc.org/’). The most common gene-fusion result by far is the *BCR::ABL1* generated by the t(9;22)(q34;q11) translocation that is specific for chronic myeloid leukemias but also occurs in Ph-positive acute leukemias. Other leukemia-relevant genes mapping to 9q34 include *SET* and *NUP214*. The differentiation between these rearrangements has important therapeutic implications. G-banding and/or FISH investigations can hint at gene fusions generated by inter-chromosomal rearrangements, *e.g.*, the t(6;9) leading to *DEK::NUP214* fusion; however, this is not the case with cryptic intra-chromosomal rearrangements such as *SET::NUP214* and *NUP214::ABL1* (all three genes map within 9q34). Furthermore, standard diagnostic FISH analyses using commercial dual fusion probes for *BCR::ABL1* (used whenever CML or ALL is suspected) may sometimes show an abnormal signal pattern for *ABL1* (mostly the presence of an extra signal) but no fusion with *BCR*. Next generation sequencing, at both DNA and RNA level, is an effective method to screen for presence of cryptic rearrangements/fusion genes. However, the commercial panels available for diagnostic purposes have specific targets and may therefore miss less common gene-fusions. Furthermore, although the screening ability of NGS technologies is indisputable; its use in standard leukemia diagnostics is still limited. We here present a combination of cytogenetic and molecular methods that can be used in a stepwise manner to identify cryptic rearrangements in neoplastic cells.

*NUP214* codes for a nuclear pore complex (NPC) component with a key role in the cell cycle, including mRNA and nuclear protein export between the nucleus and cytoplasm (2, 14). The gene has been found rearranged with several partners in leukemias. The most frequent partner is *DEK* which maps to 6p22 and codes for a chromatin-remodeling protein participating in transcription regulation (15). *DEK::NUP214* fusion (16) as a result of a seemingly balanced t(6;9)(p22;q34) is typical of AML preceded by a short myelodysplastic phase (‘https://mitelmandatabase.isb-cgc.org/’). Alternative partners for *NUP214* are *SET*, which maps to 9q34 and codes for a chromatin-remodeling protein with roles in transcription regulation (2), and the proto-oncogene *ABL1* which encodes a protein tyrosine kinase involved in many cellular processes, including cell growth and survival (17–21 ). The *SET::NUP214* fusion (22) can be generated by either the cryptic translocation t(9;9)(q34;q34) or an intra-chromosomal rearrangement such as the deletion del(9)(q34.11q34.13); it is mostly found in patients with AUL and T-ALL (1, 2, 19, 22). A *NUP214::ABL1* (23) chimeric transcript usually corresponds to a T-lineage ALL phenotype (23, 24), and patients with this fusion gene can be effectively treated with tyrosine kinase inhibitors (TKI) (25, 26). Less frequent partners for *NUP214* are the Sequestosome 1 (*SQSTM1*) gene at 5q35, involved mostly in T-ALLs (27), and the Ras-related C3 botulinum toxin substrate 1 gene (*RAC1*) at 7p22 in AML (28).

Patients with *DEK::NUP214* account for around 1% of all AML cases (1) and include both children (mean age 10 years) and adults (mean age 45 years) (29, 30); our data are in accordance with previous literature as *DEK::NUP214*fusion was present in four adults and a 15-year-old girl with AML. The exact role of *DEK::NUP214* in leukemogenesis is still unknown, but Ageberg et al. showed that it could promote phosphorylation of the eukaryotic translation initiation factor 4E (eIF4E) and activation of the mTOR signaling pathway, increasing myeloid cell proliferation (31, 32).

*SET::NUP214* was found in two patients, with ALAL-NOS and AUL, respectively, (cases 6 and 7). In both cases, FISH analysis using an *ABL1* probe showed only one fluorescent signal on 9q34/*ABL1*. The genomic imbalances detected by aCGH included loss of a genomic region at 9q34 with breakpoints inside the above-mentioned genes (*SET* and *NUP214*). RT-PCR, followed by sequencing, detected the *SET::NUP214* fusion gene in both cases. The mechanism by which *SET::NUP214* mediates leukemogenesis has not been fully elucidated, but the chimeric gene encodes a fusion protein that inhibits cell apoptosis by cytotoxic T-cells (33).

*NUP214* showed the same breakpoint (exon 18) in both types of fusion (*DEK::NUP214* and *SET::NUP214*), indicating that a similar pathogenetic mechanism, namely deregulation of the DEK and SET histone binding proteins, could be critical leading to abnormal histone hypoacetylation and silencing of genes important for lineage commitment and hematopoietic differentiation (2).

FISH analysis in case 8 and 9 using a commercial *ABL1* probe showed amplification of the gene. Graux et al. (23) were the first to show that, in such cases, the *ABL1* gene is involved in a cryptic rearrangement with *NUP214* creating from one to multiple extrachromosomal episome(s) containing the *NUP214::ABL1* fusion genes not detectable by karyotyping. RT-PCR confirmed the presence of a 9q34 rearrangement resulting in the fusion of exon 29 of *NUP214* with exon 2 of *ABL1* in case 9, and fusion of exon 31 of *NUP214* with exon 2 of *ABL1* in case 8. This is the first time that a *NUP214::ABL1* fusion gene was found together with a *PCM1::FGFR1* transcript (case 8). The patient was a 23-year-old previously healthy man diagnosed with near, early precursor T-cell leukemia (near ETP-ALL) after 2-3 weeks with night sweats, weight loss, and enlarging lymph nodes on his neck. Upon admission, anemia and thrombocytopenia were noted and leukocytes were increased to 106 x 10^9^/L (normal range 3-10).

The final diagnosis in this case was made based on examination of the bone marrow and a lymph node biopsy. Treatment with dexamethasone, vincristine, doxorubicine, asparginase, methotrexate, and mercaptopurine according to European ALL Together protocol was then started (34), whereas imatinib was added afterwards. The patient became minimal residual disease (MRD) negative and achieved complete remission on day 78. The second gene fusion in this case, *PCM1::FGFR1*, was also described by Kasbeker et al. (35 ) in a patient suffering from a rare, aggressive myeloid neoplasia with eosinophilia; treatment with *futibatinib*, an oral selective small molecule inhibitor of FGFR1-4, resulted in complete hematologic and cytogenetic remission (35). The *PCM1* gene as partner in the chimera *PCM1::JAK2* has been implicated in the pathogenesis of also other acute myeloid/lymphoid neoplasms (36) whereas rearrangements of *FGFR1* are rare (37). The use of selective TKIs can potentially affect outcomes in these patients (35, 37). Because FGFR inhibitors may be used as first-line therapy for patients with myeloid/lymphoid neoplasms with *FGFR1* rearrangement, gene-level diagnosis testing for such aberrations is clinically important (35). Finally, the detection of both *NUP214::ABL1* and *PCM1::FGFR1* fusions in the near ETP-ALL of case 8 leaves no information as to which fusion was the primary and which was secondary in this leukemia.

Aberrant expression of transcription factor oncogenes such as *TLX3* plays a major role in the pathogenesis of T-ALL and defines distinct molecular groups with prognostic significance (38). Our two T-ALL cases were also characterized by aberrant expression of *TLX3*. T-ALL patients co-expressing *TLX3* and *NUP214::ABL1* fusion transcript may belong to a subgroup of poor survival (39). However, data are still too limited to conclude.

Finally, some methodological comments on the scoring principles of aCGH analyses may be warranted. Many laboratories, including ours, follow standard guidelines/recommendations (9, 40, 41) as to the size of which imbalances to score and which not. If one sticks to a requirement of size more than 5 Mb for any imbalance to be paid attention to, one risks overlooking small but important changes. This would have been the case for patients 6, 7, 8, and 9 of our series. Therefore, when determining the scoring of imbalances, each clinical laboratory applies its own professional judgment to the specific circumstances presented by individual patients or specimens, reporting all the imbalances deemed clinically relevant; this is also fundamental to avoid false positives in case of low-quality results. Based on our experience and the results reported here, we recommend checking and reporting all imbalances, irrespective of their size. One should also note which genes are affected so as to identify different pathways leading to the same phenotypic disease, or even identify possible therapeutic approaches associated with the rearrangement of specific genes in other contexts.

In conclusion, the use of different methodological approaches - whenever possible a combination of karyotyping, FISH and molecular methods - is often necessary to detect genomic *NUP214* rearrangements in leukemic cells. The identification of a specific *NUP214* rearrangement is fundamental to choosing an optimal therapeutic strategy: by way of example, patients with *DEK::NUP214* are classified as an adverse risk group within AML and might be considered candidates for allogenic bone marrow transplantation.

## Data availability statement

The raw data supporting the conclusions of this article will be made available by the authors, without undue reservation.

## Ethics statement

The studies were conducted in accordance with the local legislation and institutional requirements. Written informed consent for participation in this study was provided by the participants’ legal guardians/next of kin.

## Author contributions

MB: Writing – review & editing, Writing – original draft, Validation, Supervision, Resources, Methodology, Formal analysis, Data curation, Conceptualization. KA: Writing – review & editing, Methodology, Formal analysis. SS: Writing – review & editing, Data curation. AL: Writing – review & editing, Data curation. LO: Writing – review & editing, Data curation. HV: Writing – review & editing, Methodology. SH: Writing – review & editing. FM: Writing – review & editing, Writing – original draft, Visualization, Supervision, Project administration, Funding acquisition, Data curation, Conceptualization.
