# Supplementary material for: NUP214 fusion genes in acute leukemias: genetic characterization of rare cases
Source: Front Oncol. 2024 Mar 20;14:1371980. doi: 10.3389/fonc.2024.1371980 (PMC10987735; doi:10.3389/fonc.2024.1371980)
Supplement: Supplementary file 1 [file Table_1.docx]

| **Case nr** | **Immunophenotype** | **FISH for *BCR::ABL1* fusion** | **FISH for *ABL1* break-apart** | **Additional genetic analysis *** |  |  |
| --- | --- | --- | --- | --- | --- | --- |
| **1** | CD45+ dim, CD34+, CD117+, HLADR+ heterogenous, CD33+ bright, CD13+ bright, CD38+, CD123+, TdT+, cyMPO+ weak, CD19+ weak, CD16-, CD11b-, CD10-, CD64 bimodal, CD35-, IREM-2-, CD14-, CD36-, CD105-, CD71+ weak, CD15-/ dim, NG2-, CD56-, CD7-, cyCD3-, CD3-, CD79a- | - | - | *FLT3* (c.2503G>T Asp835Tyr); COSM783 |  |  |
| **2** | CD45+, CD34 heterogenous, CD71+, CD117+, CD38 +dim, HLA-DR heterogenous, CD33 ++, CD13+, CD14-, CD15-, CD11b-, CD16-, CD36-, CD64-, cyMPO-, CD2-, CD3-, CD7-, CD19-, CD20-, CD79a-, CD56-, CD235a-, CD41-, Tdt- | - | - | *FLT3* (ITD) |  |  |
| **3** | CD45+, CD34+, CD117+, HLA-DR+, CD38+, CD13+, CD33+, CD71+, cyMPO+ weak, CD11b-, CD16-, CD10-, CD64-, CD35-, CD14-, IREM2-, CD36-, CD105-, CD7-/+, cyCD3-, CD19-, cyCD79a-, CD56-, Tdt- | - | - | *FLT3* (ITD) |  |  |
| **4** | CD45+dim, CD117+, CD34+(50%), HLA-DR+ heterogenous, CD13+, CD33+, CD123+ weak, CD38+, CD99+, cyMPO-/ dim, CD133+ weak CD11b-, CD36-, CD64-, CD14-, CD15 -, NG2-, CD2-, CD19-, CD7-, CD4-, CD35-, IREM2-, CD16-, CD10-, CD25-, CD41a-, CD5-, CD42b-, CD105-, CD71-, cy CD79a-, CD3-, cyCD3- | 2 *ABL1* signals (100%) | - |  |  |  |
| **5** | CD45 weak+, CD34+, CD117+, HLA-DR+, CD33+, CD13+, CD11b-, CD16-, CD10-, CD35-, CD64-, IREM2-, CD14-, CD56-, CD36- (11%+), CD105+, CD71+, CD15-, NG2-, CD2, CD19, CD7+ weak (30%), CD96+, CD123+, CD38+, CD99+, CD11a+, CD133 +, CD4-, cyMPO-/+, CD3-, cyCD3-, CD79a-, Tdt- | - | - |  |  |  |
| **6** | CD45+, CD34-, CD117+, HLA-DR-, cyMPO-, cyCD79a+ (50 %, weak), CD3-, cyCD3-, CD56-,CD13-, CD33+ (22%, weak),CD11b+ (weak), CD36-, CD64-, CD14-, CD15-, NG2-, CD2-, CD19+ (weak), CD7+ (heterogenous), CD96+ (weak), CD123+ (41 %, weak), CD38+, CD99+ (bright), CD11a+ (heterogenous), CD133+, CD4-, CD25-, CD41a-, CD5-, CD42b-, CD35, IREM2-, CD105-, CD71+(weak), CD16-, CD10-, CD20-, CD24-, -, nTdT-, CD22+ (50%, weak), CD16/CD56, TCRγδ-, CD8-, kappa-, lambda-, cykappa-, cylambda-, CD138- | 1 *ABL1* signal (20%) | - | *JAK3* (c.2570T>C (p.L857P); COSM1666992  *PHF6* (p.H149G) |  |  |
| **7** | CD45weak+, CD34+, CD117-, HLA-DR+, CD33+, CD123+, CD38+dim, CD99++, CD7++, CD13-, CD123+, CD11b dim, cyCD3-, cyCD79a-, cyMPO-,CD64-, CD14-,CD36ov-, CD15-, NG2-, CD2-, CD96-, CD11a-, CD133-, CD5-, CD2-, CD3-, CD4-, CD8-, CD16-, CD56-, CD19+, cCD22-, nuTdT-, CD10-, kappa-, lambda-, CD20-, CD42b-, CD9-, CD25-, CD41a- CD71+heterogenous, CD105-, CD64-, CD35-, CD14-, CD15-, NG2- | 1 *ABL1* signal (39%) | - | *NRAS* (c.35G>T (p.G12V); COSM566  *PHF6* (p.T208N) |  |  |
| **8** | CD45+dim, CD34+, CD7+, CD3+ weak, cyCD3+, CD4+heterogeneous, CD8, CD2heterogeneous, CD5+weak, TCR αβ-, TCRγδ+, CD48-, CD16/CD56-, CD117-, HLADR-, CD38+, CD99+, CD1a -, nuTdT+, CD19-, CD20-, CD10heterogenous, CD22-, cyCD79a-, CD24-, CD33heterogenous, CD13-, CD11b-, CD64-, CD11c-, CD14-, cyMPO-, CD13/CD33+dim | Amplification of *ABL1* (38%) | *ABL1* amplification (38%) |  |  |  |
| **9** | CD45+weak, CD7+, cyt CD3+, CD3-, CD4+ weak, CD8-, CD5+, CD99++, CD2+ weak, CD1a+, nuTdT+, TCRab-, TCR gd-, cyt MPO-, cytCD79a-, cyt CD22-, CD19-, CD10-, CD13-, CD33-, CD11c-, CD14-, CD64-, CD56- | Amplification of *ABL1* (85%) | *ABL1* amplification (85%) |  |  |  |

-: analysis not done; ITD: Internal tandem duplication

* The VariantPlex Myeloid Panel including 75 genes was used (Archer DX, Boulder, 2477 55th St #202, United States) was used to identify pathogenetic variants and sequenced on the NextSeq 2000 (Illumina, San Diego, CA, United States). Data analysis was performed following the companies recommended software using Archer Analysis 6.2.7. Annotations were based on the human reference sequence GRCh37/hg19 and pathogenicity was determined by using relevant databases: MTB Portal (Karolinska Institutet), HSMD (Qiagen), ClinVar (National Institute of Health), COSMIC (Sanger Institute) and the WHO classification.
