# Supplementary material for: NUP214 fusion genes in acute leukemias: genetic characterization of rare cases
Source: Front Oncol. 2024 Mar 20;14:1371980. doi: 10.3389/fonc.2024.1371980 (PMC10987735; doi:10.3389/fonc.2024.1371980)
Supplement: Supplementary file 2 [file Table_2.docx]

| **Table S2: Overview of the genomic imbalances** | | | | | | | |  |  |  |  |  |
| --- | --- | --- | --- | --- | --- | --- | --- | --- | --- | --- | --- | --- |
| **Sample** | **chromosome** | **gain/loss** | **copy number** | **cytogenetic location** | **Start*** | **Stop*** | **size** |  |  |  |  |  |
| **1** | 15 | gain | 3 | q11.1q13.2 | 20612553 | 31136479 | 10.59 Mb |  |  |  |  |  |
|  | 15 | loss | 1 | q13.2q15.3 | 31171498 | 44704455 | 13.62 Mb |  |  |  |  |  |
|  | 15 | gain | 3 | q15.3q26.3 | 44752208 | 102465347 | 5 57.83 Mb |  |  |  |  |  |
| **2** | No genomic imbalances |  |  |  |  |  |  |  |  |  |  |  |
| **3** | No genomic imbalances |  |  |  |  |  |  |  |  |  |  |  |
| **4** | No genomic imbalances |  |  |  |  |  |  |  |  |  |  |  |
| **5** | 16 | loss | 1 | p12.2 | 21956513 | 22415464 | 458.9 Kb |  |  |  |  |  |
| **6** | 1 | gain | 3 | p34.3p33 | 36704066 | 47697855 | 11.01 Mb |  |  |  |  |  |
|  | 1 | loss | 1 | p31.1p11.2 | 79096475 | 121350692 | 30.47 Mb |  |  |  |  |  |
|  | 3 | loss | 1 | p12.2p12.1 | 80409075 | 85791973 | 5.44 Mb |  |  |  |  |  |
|  | 5 | loss | 1 | p15.1p13.3 | 17364335 | 30672874 | 13.35 Mb |  |  |  |  |  |
|  | 5 | loss | 1 | q23.2q32 | 125658644 | 149408856 | 23.76 Mb |  |  |  |  |  |
|  | 6 | loss | 1 | q12 | 64280957 | 69364477 | 5.15 Mb |  |  |  |  |  |
|  | 8 | loss | 1 | q23.2q23.3 | 110727483 | 116425599 | 5.75 Mb |  |  |  |  |  |
|  | 9 | loss | 1 | p24.1p22.3 | 8962200 | 15479714 | 771 b |  |  |  |  |  |
|  | 9 | loss | 1 | p21.3p21.1 | 22006846 | 32607657 | 2.59 Mb |  |  |  |  |  |
|  | 9 | loss | 1 | p13.2 | 36966479 | 36966621 | 6.56 Mb |  |  |  |  |  |
|  | **9** | **loss** | **1** | **q34.11q34.13** | **131452393** | **134038370** | **10.62 Mb** |  |  |  |  |  |
|  | 11 | loss | 1 | p14.3p13 | 22303569 | 31770851 | 9.52 Mb |  |  |  |  |  |
|  | 11 | loss | 1 | p13p11.2 | 34381413 | 44077123 | 9.76 Mb |  |  |  |  |  |
|  | 12 | loss | 1 | q21.2q21.33 | 76786272 | 92449619 | 15.73 Mb |  |  |  |  |  |
|  | 13 | loss | 1 | q14.2 | 48880950 | 49050692 | 170.63 Kb |  |  |  |  |  |
|  | 13 | loss | 1 | q14.3q21.1 | 54283205 | 59490184 | 5.27 Mb |  |  |  |  |  |
|  | 13 | loss | 1 | q21.2q21.33 | 61835119 | 72906495 | 11.15 Mb |  |  |  |  |  |
|  | 13 | loss | 1 | q31.1q31.3 | 81216251 | 93523140 | 12.36 Mb |  |  |  |  |  |
|  | 14 | loss | 1 | q23.2q23.3 | 62234039 | 67511342 | 5.34 Mb |  |  |  |  |  |
|  | 21 | loss | 1 | q21.1q21.2 | 19431423 | 26689138 | 7.32 Mb |  |  |  |  |  |
| **7** | 1 | loss | 1 | p36.33p36.13 | 864428 | 16627785 | 15.83 Mb |  |  |  |  |  |
|  | 1 | gain | 3 | q32.1q44 | 198739286 | 249238072 | 50.54 Mb |  |  |  |  |  |
|  | 3 | loss | 1 | p26.3p26.2 | 64052 | 3644661 | 3.67 Mb |  |  |  |  |  |
|  | 7 | gain | 3 | p22.3p21.1 | 55649 | 16963762 | 17 Mb |  |  |  |  |  |
|  | 7 | loss | 1 | q36.1 | 149874172 | 151876948 | 2.15 Mb |  |  |  |  |  |
|  | **9** | **loss** | **1** | **q34.11q34.13** | **131462548** | **134029276** | **2.57 Mb** |  |  |  |  |  |
|  | 10 | gain | 3 | p15.3q26.3 | 139069 | 135477952 | 135.53 Mb |  |  |  |  |  |
|  | 11 | gain | 3 | q13.1q13.2 | 64579499 | 67256419 | 2.68 Mb |  |  |  |  |  |
|  | 12 | gain | 3 | q13.12 | 49432876 | 51055850 | 1.68 Mb |  |  |  |  |  |
|  | 16 | gain | 3 | p12.1p11.2 | 27934418 | 31513172 | 3.64 Mb |  |  |  |  |  |
|  | 17 | gain | 3 | q21.2q21.31 | 40055869 | 41230915 | 1.23 Mb |  |  |  |  |  |
|  | 17 | gain | 3 | q21.31 | 41232148 | 43549397 | 2.35 Mb |  |  |  |  |  |
|  | 17 | loss | 1 | q21.31 | 44188179 | 44771817 | 642.42 Kb |  |  |  |  |  |
|  | 17 | gain | 3 | q25.1q25.2 | 72182897 | 75186894 | 3.07 Mb |  |  |  |  |  |
|  | 19 | gain | 3 | p13.3 | 1736824 | 4361215 | 2.63 Mb |  |  |  |  |  |
|  | 19 | gain | 3 | p13.3 | 4366538 | 6097076 | 1.77 Mb |  |  |  |  |  |
|  | 19 | gain | 3 | p13.3p13.2 | 6611579 | 12106500 | 5.52 Mb |  |  |  |  |  |
|  | 19 | gain | 3 | p13.2p13.12 | 12191207 | 15349956 | 3.18 Mb |  |  |  |  |  |
|  | 19 | gain | 3 | p13.12p13.11 | 15365104 | 18339145 | 3.02 Mb |  |  |  |  |  |
|  | 19 | gain | 3 | p13.11p12 | 18886441 | 20048405 | 1.2 Mb |  |  |  |  |  |
|  | 19 | gain | 3 | q13.12q13.2 | 35992224 | 39654349 | 3.74 Mb |  |  |  |  |  |
|  | 19 | gain | 3 | q13.32q13.33 | 46034391 | 48185833 | 2.22 Mb |  |  |  |  |  |
|  | 19 | gain | 3 | q13.33 | 48260266 | 51359142 | 3.1 Mb |  |  |  |  |  |
|  | 22 | gain | 3 | q12.3 | 35101644 | 35676479 | 633.04 Kb |  |  |  |  |  |
|  | 22 | gain | 3 | q13.2 | 41552376 | 43651260 | 2.13 Mb |  |  |  |  |  |
| **8** | 3 | loss | 1 | q26.33 | 179373147 | 181486416 | 2.17 Mb |  |  |  |  |  |
|  | 7 | loss | 1 | q11.1q11.21 | 61081314 | 62212397 | 4.43 Mb |  |  |  |  |  |
|  | 8 | loss | 1 | p22 | 17880976 | 18400239 | 563.72 Kb |  |  |  |  |  |
|  | 8 | loss | 1 | p11.23p11.22 | 38275589 | 38767841 | 363.49 Kb |  |  |  |  |  |
|  | 9 | loss | 0 | p21.3 | 20504934 | 23231313 | 2.78 Mb |  |  |  |  |  |
|  | 9 | loss | 0 | p21.3 | 20624804 | 21974644 | 1.35 Mb |  |  |  |  |  |
|  | 9 | loss | 0 | p21.3 | 20624804 | 23231313 | 2.64 Mb |  |  |  |  |  |
|  | 9 | loss | 1 | p13.1p11.2 | 38993993 | 47199237 | 26.82 Mb |  |  |  |  |  |
|  | 9 | loss | 1 | q12q21.11 | 65639425 | 69846276 | 23.44 Mb |  |  |  |  |  |
|  | **9** | **gain** | **3** | **q34.12q34.13** | **133683984** | **134095240** | **439.84 Kb** |  |  |  |  |  |
|  | 11 | loss | 1 | p13 | 31954457 | 34697960 | 2.85 Mb |  |  |  |  |  |
|  | 14 | loss | 1 | q11.2 | 19323088 | 20420346 | 20.43 Mb |  |  |  |  |  |
|  | 15 | loss | 1 | q11.1q11.2 | 20003669 | 22378339 | 22.42 Mb |  |  |  |  |  |
| **9** | 3 | loss | 1 | p26.3 | 727753 | 1608525 | 939.22 Kb |  |  |  |  |  |
|  | 9 | loss | 0 | p21.3 | 21902774 | 24232780 | 2.39 Mb |  |  |  |  |  |
|  | **9** | **gain** | **6** | **q34.12q34.13** | **133683984** | **134075591** | **419.53 Kb** |  |  |  |  |  |
|  | 11 | loss | 1 | p13 | 32176573 | 33117912 | 1.03 Mb |  |  |  |  |  |
|  | 11 | loss | 1 | q14.1q24.2 | 85542806 | 125720535 | 40.25 Mb |  |  |  |  |  |
|  | 16 | loss | 1 | q22.1 | 67296863 | 67750789 | 498.45 kb |  |  |  |  |  |
|  | 19 | loss | 0 | q13.2 | 42751701 | 43155649 | 492.71 Kb |  |  |  |  |  |

* Based on the human reference sequence GRCh37/hg19
