# Supplementary material for: NUP214 fusion genes in acute leukemias: genetic characterization of rare cases
Source: Front Oncol. 2024 Mar 20;14:1371980. doi: 10.3389/fonc.2024.1371980 (PMC10987735; doi:10.3389/fonc.2024.1371980)
Supplement: Supplementary file 3 [file Table_3.docx]

**Table S3. Primers used for RT-PCR reactions**

| **Name** | **Sequence (5’ to 3’)** | **Gene** | **Reference sequence; Position; Exon** |
| --- | --- | --- | --- |
| DEK-1031F | GCCAATGTTAAGAAAGCAGATAGCAG | *DEK* | NM_003472.4; 993-1018; exon 8 |
| NUP214-2767R | CTGAATCACCTGGTGGATAGTCTTCA | *NUP214* | NM_005085.4; 2742-2767; exon 19 |
| DEK-1015F | GCAGCACCACCAAGAAGAATCAA | *DEK* | NM_003472.4; 1015-1037; exon 8 |
| NUP214-2615R | GATGCTGATCCCACTCCAAGTCT | *NUP214* | NM_005085.4; 2615-2637; exon 18 |
| DEK-1105F | AGAAACCCCCTACAGATGAAGAGTT | *DEK* | NM_003472.4; 1105-1129; exon 9 |
| SET-731F | TCTGATGCAGGTGCTGATGAGT | *SET* | NM_001122821.2; 731-752; exon 6 |
| NUP214-2755R | CGGAGCTGCTGAAGACTATCCA | *NUP214* | NM_005085.4; 2755-2776; exon 19 |
| NUP214-5741F | TGGGTCTGGAAACACTGGAAGAG | *NUP214* | NM_005085.4; 5741-5763; exon 31 |
| ABL1-404R | GCAATGCCGCTGAGTATCTGCTGA | *ABL1* | NM_007313.3; 594-617; exon 3 |
| PCM1-5987FW | AACAGCCTAACCCTTTGCCG | *PCM1* | NM_006197.4; 5987-6006; exon 36 |
| FGFR1-2419REV | TGGAGGCATACTCCACGATGA | *FGFR1* | NM_023110.3; 2419-2439; exon 13 |

F or FW: Forward;

R or REV: Reverse
